# Clonal dynamics underlying the skewed CD4/CD8 ratio of mouse thymocytes revealed by TCR-independent barcoding

**DOI:** 10.1038/s42003-022-03870-3

**Published:** 2022-09-05

**Authors:** Norimasa Iwanami, Malte Petersen, Dagmar Diekhoff, Thomas Boehm

**Affiliations:** 1 Department of Developmental Immunology, Max Planck Institute of Immunobiology and Epigenetics, Stuebeweg 51, D-79108 Freiburg, Germany; 2 Faculty of Medicine, Albert Ludwigs University, Breisacher Strasse 153, D-79110 Freiburg, Germany; 3 Present Address: Center for Bioscience Research and Education, Utsunomiya University, Utsunomiya, Tochigi, 321–8505 Japan; 4 Present Address: High Performance Computing & Analytics Lab, University of Bonn, Friedrich-Hirzebruch-Allee 8, D-53115 Bonn, Germany

**Keywords:** Immunogenetics, Development

## Abstract

T cell differentiation in the thymus generates CD4^+^ helper and cytotoxic CD8^+^ cells as the two principal T cell lineages. Curiously, at the end of this complex selection process, CD4^+^ cells invariably outnumber CD8^+^ cells. Here, we examine the dynamics of repertoire formation and the emergence of the skewed CD4/CD8 ratio using high-resolution endogenous CRISPR/Cas9 barcoding that indelibly marks immature T cells at the DN2/DN3 pre-TCR stage. In wild-type mice, greater clone size of CD4^+^ cells and an intrinsically greater probability of Tcr β clonotypes for pMHCII interactions are major contributors to the skewed CD4/CD8 ratio. Clonal perturbations of thymocyte differentiation following the precocious expression of a rearranged iNKT invariant TCR α chain are due to loss of Tcr β clonotypes from the CD4 lineage-committed pre-selection repertoire. The present barcoding scheme offers a novel means to examine the clonal dynamics of lymphocyte differentiation orthogonal to that using TCR clonotypes.

## Abstract (web summary)

CRSIPR-mediated barcoding of pre-TCR-expressing T cells is used to investigate the clonal dynamics of thymic T cells and reveals new fundamental understanding of how the skewed CD4/CD8 ratio arises during thymic selection.

## Introduction

More than 40 years ago, antisera to different Ly allo-antigens were shown to distinguish functionally distinct subpopulations of T cells in mice^1–3^; soon after, analogous functional subsets of human T cells were identified using monoclonal antibody reagents^4^. These studies established that the ratios of the two major subgroups, now known as CD4^+^ helper and CD8^+^ cytotoxic T cells, were fixed in a strain- and species-specific fashion, with CD4^+^ cells generally outnumbering CD8^+^ cells. Follow-up studies indicated that the skewed CD4/CD8 ratio is established in the thymus^5–8^, although several genetic and non-genetic factors impact its magnitude in the periphery^8–12^. Both cell-extrinsic^13^ and cell-intrinsic^5,14,15^ regulators were identified that favour CD4^+^ or CD8^+^ lineage commitment in the thymus. In addition to the composition of the α and β chains of the T cell receptor (TCR)^5,14,15^ asymmetric cell death during the development of CD4^+^ and CD8^+^ thymocytes^16^ has been invoked to explain at least part of the skewed CD4/CD8 ratio.

Recent deep sequencing studies of TCR repertoires have substantiated the notion that the particular composition of the αβ heterodimer contributes to CD4/CD8 lineage choices^17,18^. Here, we set out to examine the process of CD4 and CD8 differentiation using endogenous barcoding of immature thymocytes. We chose to barcode thymocytes at the DN2/DN3 stage, at the time of pre-TCR formation (i.e., after T cell receptor beta chain gene [*Tcrb*] chain gene assembly), but before a functional αβ heterodimer is generated and subsequently subjected to intrathymic selection. To this end, we used a novel CRISPR/Cas9 barcoding scheme that indelibly marks immature T cells at the X-chromosomal *Hprt* locus^19^. This strategy allowed us to follow the dynamics of the T cell repertoire irrespective of the nature of *Tcrb* clonotypes. We identify two contributors to the skewed CD4/CD8 ratio: (1) On average, CD4^+^ clones are larger than CD8^+^ clones; (2) On average, *Tcrb* clonotypes have a greater chance to be selected into the CD4^+^ lineage. We also applied this strategy to the situation of precocious expression of a rearranged iNKT invariant TCR α chain and show that the associated reduction of CD4^+^ cells is due to the selective loss of *Tcrb* clonotypes from the pre-selection repertoire of CD4^+^ cells.

## Results and discussion

### Implementation of a high-resolution barcoding system

**Fig. 1:**
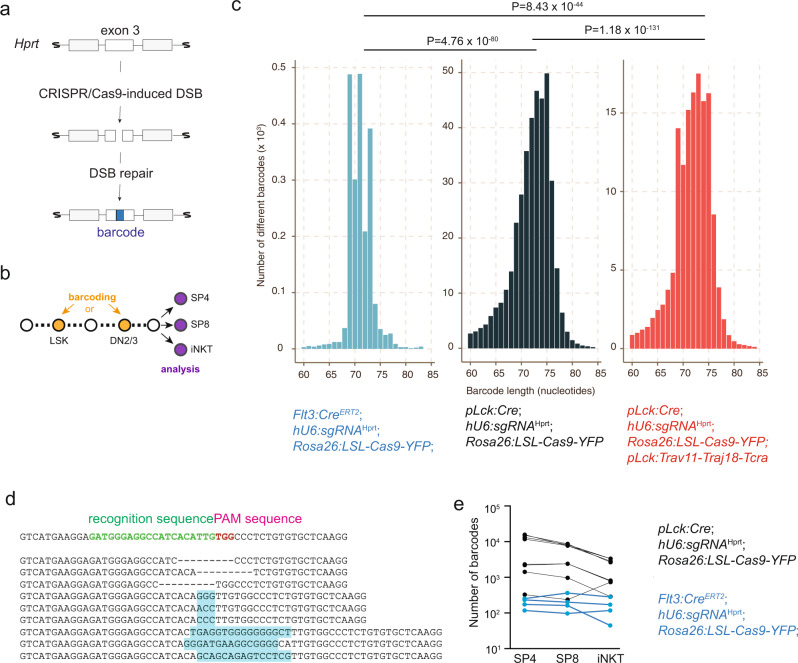
Characterization of the barcoding system. **a** Schematic illustrating the successive steps in barcode generation. **b** Schematic indicating the two stages of barcoding at the LSK bone marrow progenitor, and the DN2/3 immature thymocyte stages; the common analysis time point at the mature thymocyte stage is also indicated. **c** Size distributions of barcode sequences in mice of the indicated genotypes. The values for mean±standard deviation are, from left to right, 71.037 ± 2.337 nucleotides; 72.038 ± 3.782 nucleotides; 71.762 ± 3.602 nucleotides, respectively. P values for two-way comparisons are indicated (t-test). **d** Examples of barcode sequences with deletions (indicated by dashes) and insertions (highlighted by blue shading). The first nucleotide in the wild-type sequence corresponds to nucleotide 303, the last nucleotide corresponds to nucleotide 354 in Genbank accession number NM_013556.2. **e** Number of barcodes recovered from individual thymocyte populations in mice of the indicated genotypes.

In order to determine the clonal relationships of developing thymocytes, we used an endogenous barcoding system (Fig. 1a) that consists of three components^19^. (i) A construct for the ubiquitous expression of the *Hprt* gene-specific sgRNA (*hU6:sgRNA*^Hprt^)^19^; (ii) A conditional Cas9 expression construct (*Rosa26:LSL-Cas9-*YFP) inserted into the ubiquitously transcribed *Rosa26* locus^20^; (iii) A *pLck*:*Cre* expression construct^21^. Once the proximal *Lck* promotor (*pLck*) becomes active in DN2/DN3 thymocytes, Cre recombinase is produced, and removes a stop cassette in the *Rosa26* locus to initiate *Cas9* gene expression; the Cas9 protein forms a specific RNP complex with the ubiquitously expressed sgRNA that attacks exon 3 of the *Hprt* gene to generate double strand breaks (DSBs); error-prone repair of the DSBs leads to the formation of “scar sequences”, which serve as unique barcode identifiers for all subsequent progeny of a particular DN2/DN3 thymocyte. Owing to the inevitable delay in Cas9 activation, it is formally possible that barcode generation continues into the DN4 stage, or perhaps even into the early DP stages; note, however, that positive selection only begins in the DP stage, suggesting that most barcodes are fixed before selection begins. In male *hU6:sgRNA*^Hprt^; *Rosa26:LSL-Cas9-YFP*; *pLck*:*Cre* triple-transgenic mice, each cell possesses a single barcode, since the *Hprt* gene is located on the X chromosome; it can be read out at the DNA level after amplification of exon 3 sequences.

**Fig. 2:**
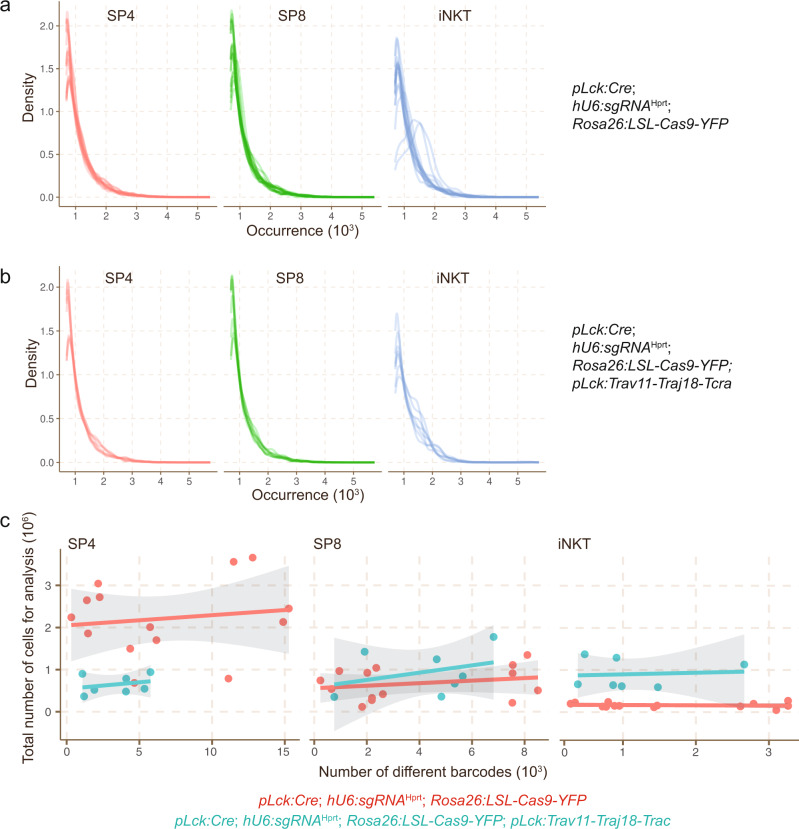
Frequency of barcodes. **a**, **b** Frequency distributions of barcodes recovered from individual thymocyte populations in mice of the indicated genotypes. **c** Number of barcodes recovered from individual thymocyte populations in mice of the indicated genotypes as a function of cell input.

Several features of the barcoding system are notable. The overall size distributions of barcodes appear to depend on the target cell (as illustrated here for LSK lymphoid progenitors in the bone marrow, and DN2/3 thymocytes, respectively [Fig. 1b]), possibly reflecting cell type-specific differences in DSB repair processes; and on the outcome of intra-thymic selection processes (Fig. 1c). Barcodes often contain deletions, but also insertions of non-templated sequences (Fig. 1d). The overall sequence complexity of barcode sequences depends on the target cell population. For instance, the *Flt3:Cre*^ERT2^ transgene is active in LSK bone marrow progenitors^22^, whereas *pLck:Cre* is active in DN2/DN3 thymocytes^21^. Therefore, when barcodes are read out in mature thymocyte subsets (CD4 single-positive [SP4] and CD8 single-positive [SP8] cells) in mice carrying the *Flt3:Cre*^ERT2^ transgene, only a few hundred different barcodes are detectable, whereas tens of thousands are found in case of the *pLck:Cre* transgene (Fig. 1e); this outcome mirrors the large difference in the numbers of cells in the target populations (~2 × 10^2^ for LSK; ~2 × 10^5^ for DN2/DN3 [Ref. ^22^]). Moreover, we found that the efficiency of barcode generation varies among individuals of the same genotype (Fig. 1e); however, despite the variations in absolute numbers of barcodes in an individual, their relative distribution among different lymphocyte lineages remains generally constant, attesting to stable trajectories of differentiation from progenitor cells (Fig. 1e). Lastly, as seen with other barcoding schemes^23^, we found that the generative probabilities of particular barcodes vary. Whereas some barcodes are generated at high frequency, the majority of barcodes are rare; this pattern is seen irrespective of the genotype of mice (Fig. 2a, b; see below for detailed description of mouse strains). This non-uniform generative probability tends to reduce the resolution of clonal analysis, since cells eventually carrying the same barcode may have originated from distinct scarring events. Note that, since DN3 cells proliferate considerably^24^ after they have become indelibly marked, many mature thymocytes carry the same barcode. This is an important feature underlying the present study, because it makes the analysis of barcodes largely insensitive to the precise number of cells used for the analysis; this is apparent from the flat regression lines demonstrating that the numbers of detected barcodes do not correlate to the numbers of cells used for the analysis (Fig. 2c).

### Barcode diversity in mature thymocytes

Our first experiment addressed the relationship between cell numbers and barcodes in SP4 and SP8 thymocytes, respectively. SP4 and SP8 cell numbers both decline with age (Supplementary Fig. 1a), commensurate with the age-related reduction of thymopoietic activity of the thymus^25^. The number of barcodes represented in the SP4 population is greater than that in the SP8 population (Supplementary Fig. 1b). Clone sizes (numbers of cells per barcode) in SP4 and SP8 populations tend to decline over time (Supplementary Fig. 1c), indicative of reduced proliferative capacity of thymocytes and/or impaired efficiency of positive selection.

**Fig. 3:**
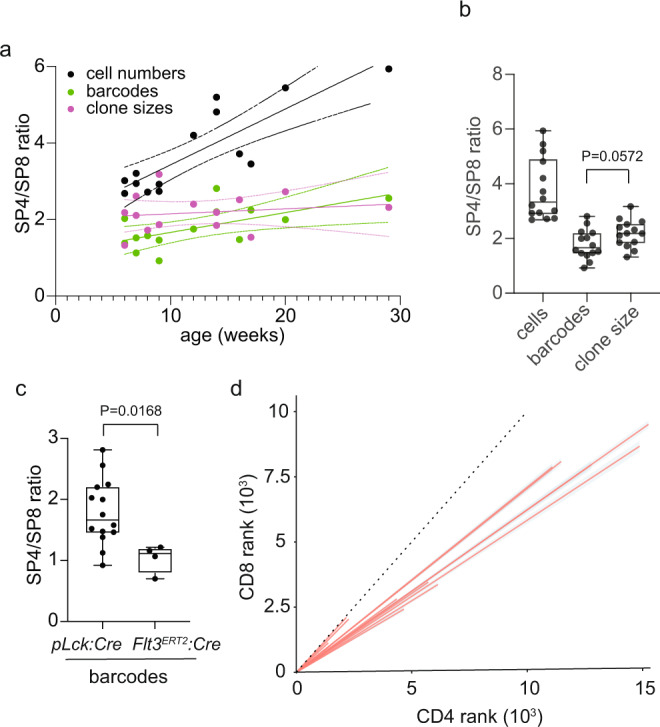
Structure of thymocyte populations. **a** Age-dependent trends of cell and barcode numbers and clone sizes expressed as SP4/SP8 ratio to normalize for inter-individual differences in barcoding efficiency as a function of age. Solid line, linear regression; dashed lines, 95% confidence interval. **b** Aggregated values of parameters shown in a. Box plots show quantiles 25 and 75 and mean (line) as well as the total range (whiskers); t-test, two-tailed (*n* = 14; P value is indicated). **c** Barcodes recovered from thymocyte populations barcoded at the DN2/DN3 stage of intrathmyic differentiation (*pLck:Cre*; *n* = 12) and at the LSK stage of lymphoid differentiation in the bone marrow (*Flt3*:*Cre*^ERT2^; *n* = 4). The difference between the populations is significant (t-test, two-tailed; *P* value is indicated). Box plots show quantiles 25 and 75 and mean (line) as well as the total range (whiskers). **d** Rank-rank correlation of shared barcodes in SP4 and SP8 thymocytes. The dotted line indicates a 1:1 correlation.

Given the inter-individual differences in barcode numbers, we calculated the SP4/SP8 ratios per mouse in order to comparatively evaluate these general trends. Over the course of the first 6 months of life, the average ratio of SP4 and SP8 cell numbers increases (Fig. 3a); when averaged over this time window, it is equivalent to 3.78 ± 1.13 (mean ± S.E.M.) (Fig. 3b), reflecting the known higher numbers of SP4 lineage cells. By contrast, the SP4/SP8 barcode ratio is stable over time (Fig. 3a), and amounts to 1.79 ± 0.54 (mean ± S.E.M.) (Fig. 3b). This result suggests that only just over half as many barcodes are found in the SP8 lineage than in the SP4 lineage during the differentiation process from DN2/DN3 to mature SP stages. The discrepancy between cell and barcode numbers translates into a SP4/SP8 clone size ratio of 2.18 ± 0.5 (mean ± S.E.M.) cells (Fig. 3b). These data identify clone number and clone size as approximately equal contributors to the skewed SP4/SP8 ratio in thymocyte populations.

### Lineage bias of *Tcrb* clonotypes

Next, we explored the basis for the skewed SP4/SP8 barcode ratio. In our scheme, barcodes are induced at the DN2/DN3 stage, and therefore become associated with particular *Tcrb* clonotypes, which are independently generated at the same stage of differentiation. Hence, a barcode ratio of ~1.8 indicates that, on average, any Tcr β clonotype is about twice as likely to contribute to an MHCII-compatible TCR αβ heterodimer expressed by SP4 cells than to an MHCI-compatible TCR αβ heterodimer expressed by SP8 cells. This result implies the presence of a strong bias of particular *Tcrb* clonotypes for either CD4 or CD8 lineages, confirming recent findings^17,18^. As a control, we examined the SP4/SP8 barcode ratio in thymocytes arising from progenitors indelibly marked in the bone marrow at haematopoietic precursor stages. By design, *Flt3:Cre*^ERT2^-induced barcodes are generated in a comparatively small number of precursor cells and are not associated with particular *Tcrb* clonotypes, which are generated much later during intra-thymic differentiation. Rather, because of the high proliferation rates of precursors before they reach the DN2/DN3 stage (in the order of at least 10 cell divisions^26^), many different *Tcrb* clonotypes eventually share the same barcode. The random distribution of *Tcrb* clonotypes among the comparatively small number of barcodes represented in SP4 and SP8 cells precludes the detection of selection bias, as indicated by a SP4/SP8 barcode ratio of 1.04 ± 0.12 (mean ± S.E.M.) (Fig. 3c).

Returning to the structure of *pLck:Cre*-induced barcode repertoires in SP4 and SP8 thymocytes, we identified and ranked shared barcodes in SP4 and SP8 populations according to their frequencies. If each barcode-associated Tcr β clonotype has an equal chance of appearing in the resultant SP4 and SP8 populations, the rank-rank correlation plot of barcodes should have an inclination of approximately 1. If, however, particular Tcr β clonotypes are incompatible with selection into either CD4 or CD8 lineages, a certain fraction of barcodes would disappear from the final SP4 and SP8 populations, respectively. In SP4/SP8 comparisons, a greater net loss of barcodes in the SP4 population would result in an inclination >1, a greater loss in SP8 cells would result in an inclination <1; the final inclination would reflect the relative contributions of these two opposing tendencies. Since only a small fraction of barcodes appears with high frequencies, the inclinations of rank-rank correlations are shaped by barcodes that are in the mid- to low-frequency range of generation probabilities; thus, our analysis strategy minimizes the impact of frequent barcodes, which are likely associated with many *Tcrb* clonotypes and hence poorly record *Tcrb*-related selection events. In wild-type mice, the inclinations for SP4/SP8 rank correlations of thymocytes in individual mice are significantly smaller than 1 (0.70 ± 0.04; mean±S.E.M.; *P* < 0.0001, single sample t-test; *n* = 14; hypothetical population mean = 1) (Fig. 3d). This indicates that during differentiation, the CD8 lineage suffers a greater loss of clonotypes from the original pool of DN2/DN3 clonotypes than the CD4 lineage. In sum, analysis of TCR repertoires at the clonal level underscores the differential compatibility of Tcr β clonotypes for selection by pMHCI and pMHCII complexes. It is instructive to compare our results with data obtained with *Tcrb* transgenic mice; whenever a significant lineage bias was observed, it correlated with the origin of the *Tcrb* clonotype. In transgenic mice expressing *Tcrb* chains of CD4^+^ cells^27–29^, a preference for the CD4 lineage was observed; conversely, in mice expressing *Tcrb* clonotpes derived from CD8^+^ cells, the lineage bias tended to favour the development of CD8^+^ cells^30–32^. To the best of our knowledge, the effect of the expression of lineage-specific *Tcrb* clonotypes has not been tested for their effect on iNKT development. However, since iNKT-specific TCR αβ heterodimers are composed of many different *Tcrb* clonotypes, we hypothesize that such *Tcrb* clonotypes could also be found expressed in SP4 and SP8 cells.

### Population structure in *pLck:Trav11-Traj18-Trac* transgenic mice

The high-resolution barcoding system described above afforded us with an unprecedented opportunity to examine the changes in population structure in TCR transgenic mice. To illustrate the value of this approach, we chose to explore thymocyte differentiation in mice precociously expressing a rearranged *Tcra* gene. In the present case, we employed a *pLck:Trav11-Traj18-Trac* transgene that encodes the canonical TCR α chain of iNKT cells, Vα14 Jα18 Cα^33–37^. In this constellation, the iNKT-specific TCR α chain is precociously expressed on DN2/3 thymocytes, concurrently to the endogeneous TCR β chains, but prior to endogenous TCR α chains. Thus, the initial population of TCR αβ heterodimers is formed with transgenic TCR α chains and a diverse array of endogenous TCR β chains. We presume that this saturates the iNKT differentiation pathway, but does not exclude the possibility of subsequently replacing the transgenic TCR α chain by endogenous TCR α chains in a kind of receptor editing process for those TCR αβ pairs, which do not bind to iNKT-type ligands and thus cannot enter the iNKT differentiation pathway. However, it is reasonable to assume that the TCR α chain replacement is not quantitative, and we hypothesize that many of the remaining TCR αβ heterodimers are unable to bind to pMHCII complexes, contributing to the skewed representation of *Tcrb* clonotypes in the SP4 and SP8 cells. In this regard, we note that the CD1 molecule, which presents the iNKT-related ligands, is a non-classical MHCI molecule^38^; the invariant α chain may therefore be more prone to contribute to binding to classical pMHCI complexes than to pMHCII complexes.

**Fig. 4:**
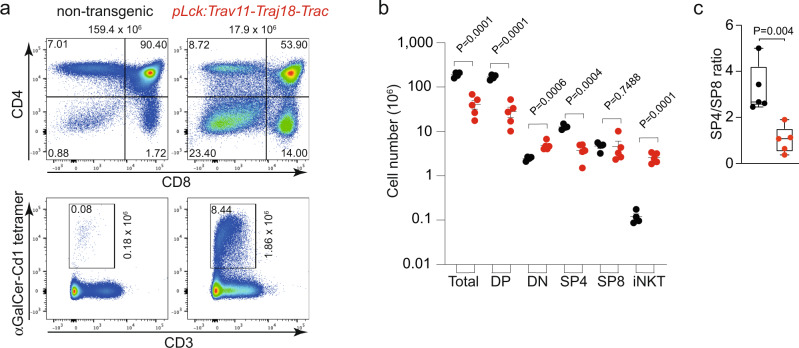
Altered thymocyte differentiation in *pLck:Trav11-Traj18-Trac* transgenic mice. **a** Representative flow cytometric profiles of non-transgenic wild-type and transgenic thymocytes of 4-week-old mice. The total numbers of thymocytes are indicated at the top of the upper panels; the proportions of individual thymocyte populations are indicated in the respective quadrants. In transgenic mice, the absolute number of iNKT cells (CD3^+^αGalCer-CD1 tetramer^+^; boxed with indicated proportions) is increased; absolute numbers are given next to the quadrant marking iNKT cells. **b** Numbers of thymocyte populations in non-transgenic wild-type (black; *n* = 4) and transgenic (red; *n* = 5) mice (mean ± s.e.m.; t-test, two-tailed; *P* values are indicated). **c** Reduced SP4/SP8 ratios in transgenic mice (red). The difference between the populations is significant (t-test, two-tailed; *n* = 5 for both genotypes; P value is indicated). Box plots show quantiles 25 and 75 and mean (line) as well as the total range (whiskers).

In *pLck:Trav11-Traj18-Trac* transgenic mice, the fractions of CD4/CD8-double negative (DN), CD4/CD8-double positive (DP), and SP4 and SP8 thymocytes are significantly altered (Fig. 4a, b), as noted in previous experiments using a similar construct^33^. Note that the extent of lineage bias depends on the type of promotor used to express the transgene; when the expression of *Trav11-Traj18-Trac* occurs at a later stage of thymocyte differentiation, the lineage bias is minimal^39^. In our transgenic mice, whereas the number of SP8 cells remains unchanged, the numbers of DP and SP4 cells are reduced (Fig. 4b). As a result, the SP4/SP8 ratio significantly drops to ~1 (Fig. 4c); by contrast, staining of thymocytes with the αGalCer–CD1d tetramer indicates a ~10-fold increase of iNKT cells (Fig. 4a, b), as expected^33^.

### Distribution of barcodes in mature transgenic thymocyte

In order to explore the mechanistic underpinnings of the skewed SP4/SP8 ratio, we combined the *pLck:Trav11-Traj18-Trac* transgene with the tri-partite barcoding system. For simplicity, we henceforth refer to the barcoding configuration (*pLck:Cre*; *hU6:sgRNA*^Hprt^; *Rosa26:LSL-Cas9-YFP*) as wild-type, and to mice additionally expressing the *pLck:Trav11-Traj18-Trac* construct (*pLck:Cre*; *hU6:sgRNA*^Hprt^; *Rosa26:LSL-Cas9-YFP*; *pLck:Trav11-Traj18-Trac*) as transgenic.

**Fig. 5:**
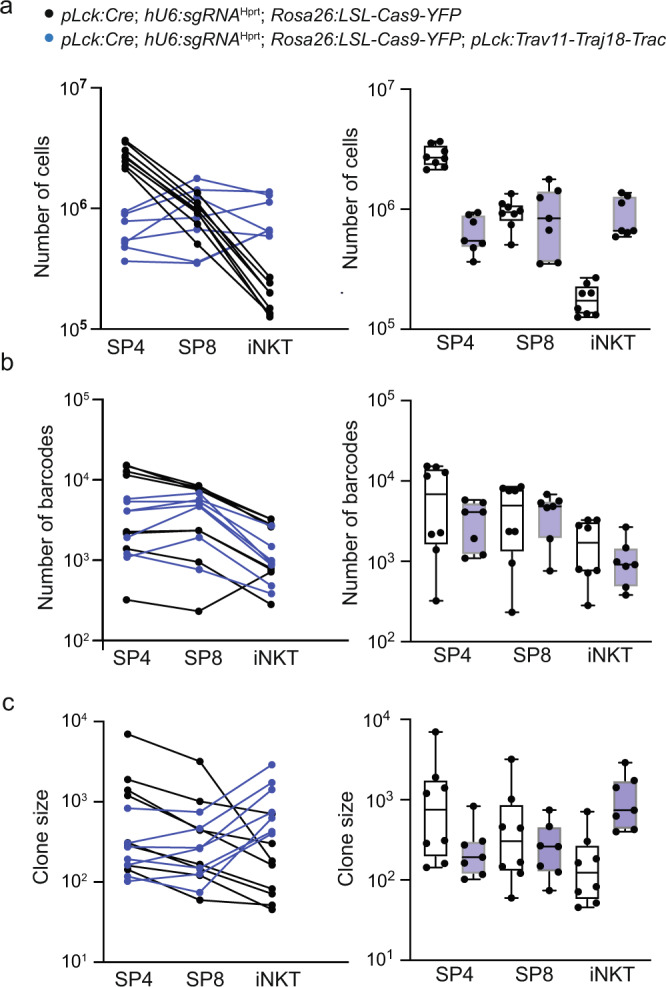
Altered thymocyte population structure in *pLck:Trav11-Traj18-Trac* transgenic mice. **a** Absolute cell numbers of thymocyte populations in mice of the indicated genotypes; lines connect data for individual mice to indicate stability of differentiation trajectories (left panel). The right panel is a summary presentation of data. **b** Absolute barcode numbers of thymocyte populations in mice of the indicated genotypes to indicate stability of differentiation trajectories irrespective of barcoding efficiency (left panel). The right panel is a summary presentation of data. **c** Clone sizes of thymocyte populations in mice of the indicated genotypes (left panel). The right panel is a summary presentation of data. In this experiment, mouse cohorts of approximately the same age (5–12 weeks) were included. *pLck:Cre;hU6:sgRNA*^Hprt^*;Rosa26:LSL-Cas9-YFP* (*n* = 8); *pLck:Cre;hU6:sgRNA*^Hprt^*;Rosa26:LSL-Cas9-YFP;pLck:Trav11-Traj18-Trac* (*n* = 7). In **a**–**c**, box plots show quantiles 25 and 75 and mean (line) as well as the total range (whiskers).

The introduction of the three transgenes of the barcoding system did not alter the composition of the thymocyte populations further; SP4 cells are reduced in the quadruple transgenic mice, SP8 cells are unchanged, and the iNKT population increases (Fig. 5a). Although the absolute numbers of detectable barcodes differ from individual to individual, we noted a trend towards more restricted barcode repertoires in SP4 cells in the transgenic situation; in 6/7 wild-type mice, SP8 cells exhibit fewer barcodes than SP4 cells, whereas this ratio is reversed in transgenic mice (2/7 mice) mice (Fig. 5b), commensurate with the changes in absolute cell numbers. By contrast, although the number of iNKT cells increases sharply in the transgenic situation, the number of barcodes is (with one exception) always lower than the numbers of barcodes in SP4 and SP8 cells (Fig. 5b), indicating that iNKT cells induced in the transgenic situation have a higher clone size (Fig. 5c).

**Fig. 6:**
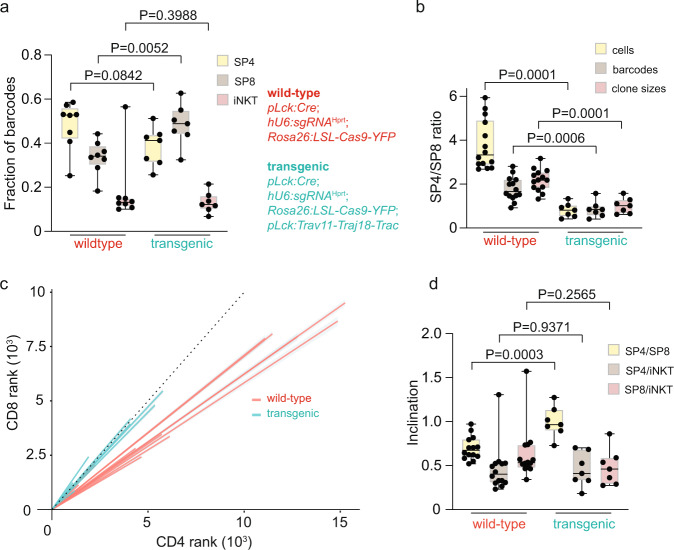
Loss of CD4-lineage committed thymocytes in *pLck:Trav11-Traj18-Trac* transgenic mice. **a** Fractions of barcodes in individual thymocyte populations of the indicated genotypes. Each data point represents one mouse (t-test, two-tailed; *pLck:Cre;hU6:sgRNA*^Hprt^*;Rosa26:LSL-Cas9-YFP* (*n* = 8); *pLck:Cre;hU6:sgRNA*^Hprt^*;Rosa26:LSL-Cas9-YFP;pLck:Trav11-Traj18-Trac* (*n* = 7); P values are indicated). **b** SP4/SP8 ratios for the indicated parameters and genotypes; t-test, two-tailed (*pLck:Cre;hU6:sgRNA*^Hprt^*;Rosa26:LSL-Cas9-YFP* (*n* = 14); *pLck:Cre;hU6:sgRNA*^Hprt^*;Rosa26:LSL-Cas9-YFP;pLck:Trav11-Traj18-Trac* (*n* = 7); P-values are indicated). **c** Rank-rank correlations of shared barcodes in SP4 and SP8 thymocytes for the indicated genotypes. Data for genotype *pLck:Cre;hU6:sgRNA*^Hprt^*;Rosa26:LSL-Cas9-YFP* are taken from Fig. 3d. **d** Summary of rank-rank correlations for the indicated comparisons of thymocyte populations for the two genotypes; t-test, two-tailed (*pLck:Cre;hU6:sgRNA*^Hprt^*;Rosa26:LSL-Cas9-YFP* (*n* = 14); *pLck:Cre;hU6:sgRNA*^Hprt^*;Rosa26:LSL-Cas9-YFP;pLck:Trav11-Traj18-Trac* (*n* = 7); P values are indicated). In **a**, **b**, **d**, box plots show quantiles 25 and 75 and mean (line) as well as the total range (whiskers).

In order to account for inter-individual differences, we calculated the fractions of barcodes that are recovered in the three different cell populations of each mouse. In transgenic animals, a relative increase in barcodes is found for the SP8 population (*P* = 0.0052; t-test, two-tailed), which was accompanied by a trend towards smaller fractions of barcodes in SP4 cells (*P* = 0.0842; t-test, two-tailed) (Fig. 6a).

The substantial re-configuration of mature SP4 and SP8 thymocyte populations in the presence of a precociously expressed rearranged *Tcra* gene suggests that the canonical iNKT TCR α chain biases the resulting TCR αβ repertoire against selection by peptide/MHCII complexes. This conclusion is reflected in the substantially reduced SP4/SP8 ratios of barcode numbers in the transgenic animals (Fig. 6b).

Compared to the wild-type situation, rank-rank-correlations of barcodes in transgenic mice (Fig. 6c) exhibit significantly increased inclinations for SP4-SP8 correlations (0.99 ± 0.07; mean ± SEM; *P* < 0.001; t-test, two-tailed) (Fig. 6d). Given that the number of barcodes tends to be smaller in transgenic SP4 cells, but remains constant in SP8 cells (Fig. 5b), the most parsimonious explanation for this observation is that the transgenic Vα14Jα18Cα chain is an unfavourable partner for many TCR β clonotypes that are normally associated with SP4 cells. As a result, such clonotypes are lost from the pool of mature SP4 cells, substantially reducing the physiological bias favouring the CD4 lineage.

Collectively, these results suggest that the balanced SP4/SP8 ratio in the transgenic situation appears to be due to a combination of diminished compatibility of SP4-biased clonotypes with the Vα14Jα18Cα chain, and reduced clone size in the SP4 lineage (Fig. 5c), perhaps as a result of poor pMHCII complex binding affinity.

With respect to the iNKT cells, we found that the fraction of barcodes associated with this cell type remains unchanged in the transgenic situation (Fig. 6a). As a result, the increase in the number of iNKT cells induced by the expression of the transgene is associated with a larger clone size (Fig. 5c), likely because the iNKT-specific TCR is formed ectopically in immature T cells with inherently higher proliferative capacity.

## Conclusions

Previously, deep sequencing of *Tcrb* clonotypes has been used to explore the developmental relationships among mature αβ T cell lineages. The present report presents an orthogonal strategy to track the outcome of T cell development in the thymus, both in the wild-type situation and under conditions of transgenic manipulation. Unlike TCR-related clonotypes, the barcodes generated in the *Hprt* gene at a specific developmental stage are not subject to direct selection via the TCR and hence provide an independent means to follow the developmental trajectories of differentiating thymocytes. Here, we have chosen the *pLck:Cre* driver to indelibly mark immature thymocytes. However, since barcodes can be introduced in any cell type or stage of differentiation for which a suitable *Cre* line is available, our strategy is applicable to other experimental circumstances as well.

The present results illuminate the basis of the skewed ratio of SP4/SP8 cells that is observed in wild-type mice. In addition to differential susceptibility to cell death^16^ and a distinct lineage bias of *Tcra* (as discussed in the Introduction and presented here) and *Tcrb* variable genes^5^, we identify differential clone sizes in the CD4 and CD8 lineages as an additional contributor to the skewed composition of the mature thymocyte populations. It is conceivable that the differential duration of TCR signalling (rather than signalling strength) during positive selection of immature cells^40^ contributes to the difference in clone sizes of SP4 and SP8 cells. Our analysis also provides insight into the fate of developing T cells under conditions of precocious expression of the invariant TCRα chain that is characteristically found in the antigen receptor of type I NKT cells (iNKT). We find that in the transgenic situation the numbers of barcodes (i.e. *Tcrb* clonotypes) in SP8 and iNKT cells remain constant; this observation suggests that the precocious provision of the invariant Vα14Jα18Cα chain does not appreciably contribute to the pool of pMHCI-selectable αβ TCR heterodimers other than those that are characteristically found in iNKT cells. At the same time, many of the Vα14Jα18Cα/β heterodimers that are formed as a result of the competition between endogenous and transgenic TCR α chains cannot be efficiently selected by pMHCII complexes, explaining the reduction of clonotype numbers and clone sizes in the CD4 lineage.

## Methods

### Animals

Transgenic mice were generated on an FVB/N background (FVB/N-tg(hU6-sgRNA-Hprt)1^Tbo^/Mpie) and subsequently backcrossed to a C57BL/6J background. The *pLck:Trav11-Traj18-Trac* construct was assembled by standard cloning techniques and is composed of the following sequences: proximal *Lck* gene promotor (nucleotide 163,417 to nucleotide 159,952 in Genbank accession number AL606921.6); *Trav11* gene cDNA sequences (nucleotide 12 to nucleotide 834 in Genbank accession number M14506.1); human growth hormone 3´- sequences (nucleotide 2596 to nucleotide 4107 in Genbank accession number KR632635.1, directly followed by nucleotide 544 to nucleotide 1169 in Genbank accession number KU665646.1). The *Flt3:Cre*^ERT2^ mouse strain has been described^22^; Cre activity was induced at the age of 4 weeks by a single i.p. injection of 3 mg tamoxifen in 0.15 mL sun flower oil (Sigma) and cells were harvested 4–5 weeks later. Mice were kept in the animal facility of the Max Planck Institute of Immunobiology and Epigenetics under specific pathogen-free conditions. All animal experiments were performed in accordance with the relevant guidelines and regulations, approved by the review committee of the Max Planck Institute of Immunobiology and Epigenetics and the Regierungspräsidium Freiburg, Germany (licence 35-9185.81/G-15/35).

### Flow cytometry

Thymocyte suspensions were prepared by mechanical liberation, best achieved by gently pressing thymic lobes through 40-μm sieves^41^; subsequent analytical flow cytometry was carried out using Fortessa instruments (Dako Cytomation-Beckman Coulter) using the following antibodies: FITC-conjugated anti-CD4 (clone GK1.5; BioLegend; dilution 1:1000); PE-conjugated anti-CD8 (clone Ly-2; eBioscience; dilution 1:800); PerCP-Cy5.5-conjugated anti-CD3ε (clone 145-2C11; Biolegend; dilution 1:100); APC-conjugated anti-CD19 (clone MB19-1; eBioscience; dilution 1:100); PE-conjugated αGalCer-CD1d Tetramer (Proimmune; dilution 1:400). For preparative flow cytometry with a BD Aria III instrument, the following antibodies were used: APC-Cy7-conjugated anti-CD4 (clone GK1.5; Biolegend; dilution 1:1000); FITC-conjugated anti-CD8 (clone 53.6.7; eBioscience; dilution 1:1000); PE-conjugated αGalCer-CD1d Tetramer (Proimmune; dilution 1:400); APC-conjugated anti-TCR (clone H57-597; eBioscience; dilution: 1: 200); PerCP-Cy5.5-conjugated anti-TCRgammadelta (clone eBioGL3; eBioscience; dilution: 1: 300).

### Barcode determination

DNA was isolated from sorted cells from each animal, and the region of exon 3 of the *Hprt* gene amplified using the following primers: 5’-ACACTCTTTCCCTACACGACGCTCTTCCGATCTTTCATAGAGACAAGGAATGTGTCC-3’ (forward, P5-DD302) and 5’-GTGACTGGAGTTCAGACGTGTGCTCTTCCGATCTAGTTGATTATGTAGCATAGTTTGCACAAG-3’ (reverse, P7-DD305). Libraries were sequenced at a depth of ~250,000 reads per sample on the MiSeq sequencing system (2x300bp). We extracted the nucleotide sequences situated between 5´- AGGACTGAAAGACTTG and 5´-TCTTTGCTGACCTGCTGG (corresponding to nucleotides 279 to 294 and nucleotides 367 to 384 in Genbank accession number NM_013556.2) and defined these subsequences as barcodes.

### Barcode analysis

Barcodes supported by 5 or more reads were kept for further analysis, removing 83.84% of low-quality reads. The lengths of these high-quality barcode sequences are approximately normally distributed; hence, we further filtered the dataset for sequences with a sequence length of more or less than 1.5 standard deviations (s.d. = 7.85) from the median length of 72 nt. At the end of this two-step filtering process, a total of 533,187 barcodes (corresponding to 13.7% of the unfiltered data) were retained. Summary statistics of the barcode length distributions in nucleotides (nt) are as follows: (1) *Flt3-Cre*^ERT2^ driver: 2,164 barcodes; minimal length, 60 nt; maximal length, 83 nt; median length, 71 nt (median absolute deviation, 2.965 nt); mean length, 71.037 nt (s.d., 2.337 nt; s.e.m., 0.05 nt). (2) *pLck-Cre* driver without *Tcra* transgene: 387,555 barcodes; minimal length, 60 nt; maximal length, 84 nt; median length, 73 nt (median absolute deviation, 2.965 nt); mean length, 72.038 nt (s.d., 3.782 nt; s.e.m., 0.006 nt). (3) *pLck-Cre* driver with *Tcra* transgene: 143,468 barcodes; minimal length, 60 nt; maximal length, 84 nt; median length, 72 nt (median absolute deviation, 2.965 nt); mean length, 71.762 nt (s.d., 3.602 nt; s.e.m., 0.01 nt). We tested for significant differences in barcode nucleotide length distributions between the three genotypes analyzed here using a t-test, implemented in the R package rstatix^42^. To investigate the relationship in barcode frequencies between the cell types of the same mouse, we plotted the frequency rank of barcodes (the position in the list of descending frequency) in one cell type against the frequency rank of the same barcode in another cell type. We computed the linear regression lines to delineate regression line “bundles” to compare the barcode frequency rank correlations in a two-way comparison. Using the fit points from these regressions, we compared the slopes between mice groups with the emtrends function from the emmeans R package^43^.

### Statistics and reproducibility

Two tailed t-tests were used to determine the significance levels of the differences between the means of two independent samples, considering equal or unequal variances as determined by the F-test. For multiple tests, the conservative Bonferroni correction was applied. For all analyses, several biological replicas were studied; the number of replicas is indicated in the figure and/or legends.

### Reporting summary

Further information on research design is available in the Nature Research Reporting Summary linked to this article.

## Supplementary information

Supplementary Information

Description of Additional Supplementary Data

Supplementary Data

Reporting Summary

## Data Availability

Source data are provided with this paper in Supplementary Data 1.
