# Supplementary material for: Clonal dynamics underlying the skewed CD4/CD8 ratio of mouse thymocytes revealed by TCR-independent barcoding
Source: Commun Biol. 2022 Sep 5;5:911. doi: 10.1038/s42003-022-03870-3 (PMC9445074; doi:10.1038/s42003-022-03870-3)
Supplement: Supplementary file 1 — Supplementary Information [file 42003_2022_3870_MOESM1_ESM.pdf]

Supplementary Material

Clonal dynamics underlying the skewed CD4/CD8 ratio of mouse thymocytes revealed by TCR-independent barcoding

Norimasa Iwanami, Malte Petersen, Dagmar Diekhoff, Thomas Boehm

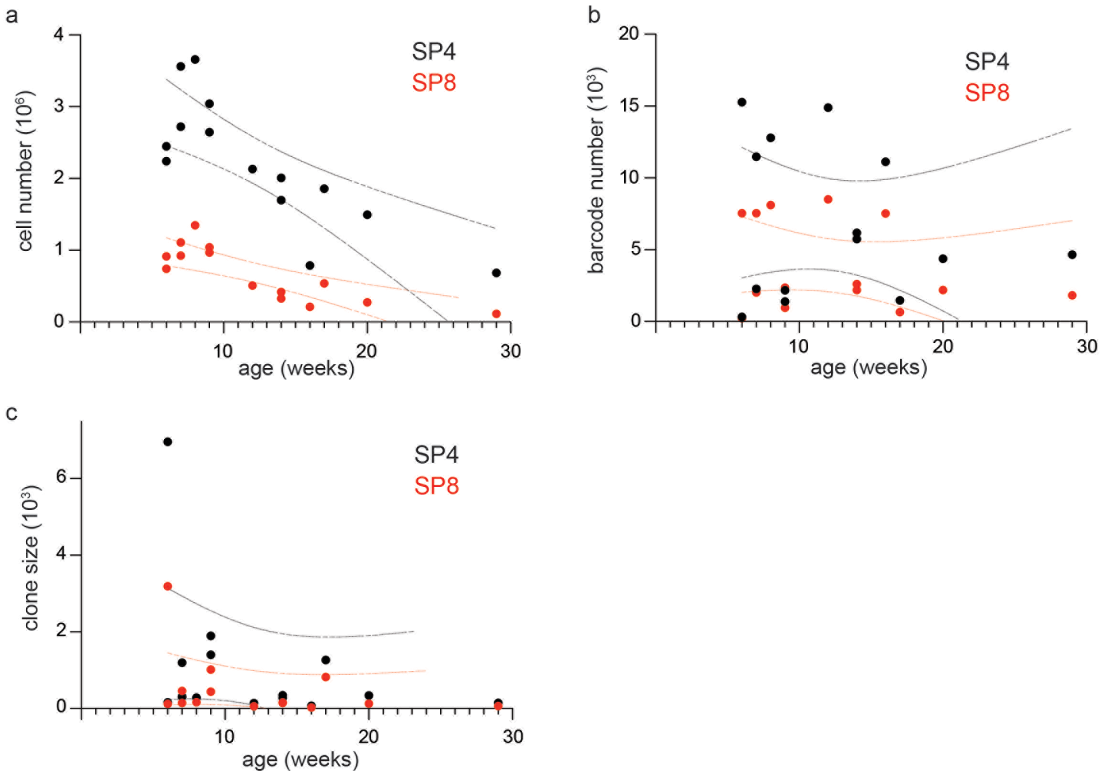

Supplementary Fig. 1 Age-dependent changes in thymocyte populations. **a** Absolute numbers of thymocytes. **b** Absolute numbers of barcodes. **c** Clone sizes. Dashed lines, 95% intervals of linear regressions.
