# Supplementary material for: Clonal dynamics underlying the skewed CD4/CD8 ratio of mouse thymocytes revealed by TCR-independent barcoding
Source: Commun Biol. 2022 Sep 5;5:911. doi: 10.1038/s42003-022-03870-3 (PMC9445074; doi:10.1038/s42003-022-03870-3)
Supplement: Supplementary file 2 — Description of Additional Supplementary Data [file 42003_2022_3870_MOESM2_ESM.docx]

**Description of Additional Supplementary Files**

**File name:** Supplementary Data 1 Sheet 1

**Description:** Source_Data_Fig_1(left)

**File name:** Supplementary Data 1 Sheet 2

**Description:** Source_Data_Fig_1c(middle)_2a_3d_6c

**File name:** Supplementary Data 1 Sheet 3

**Description:** Source_Data_Fig_1c(right)_2b_6c

**File name:** Supplementary Data 1 Sheet 4

**Description:**Source_Data_Fig_1e

**File name:**  Supplementary Data 1 Sheet 5

**Description:**Source_Data_Fig_2c

**File name:** Supplementary Data 1 Sheet 6

**Description:** Source_Data_Fig_3

**File name:** Supplementary Data 1 Sheet 7

**Description:** Source_Data_Fig_4

**File name:** Supplementary Data 1 Sheet 8

**Description:** Source_Data_Fig_5

**File name:** Supplementary Data 1 Sheet 9

**Description:** Source_Data_Fig_6Supplementary Data 1 Sheet 10 Source_Data_Supp_Fig_1
